# ﻿*Carexqingyuanensis* (Cyperaceae), a new species from Guangdong, China

**DOI:** 10.3897/phytokeys.241.117734

**Published:** 2024-04-25

**Authors:** Ya-Li Li, Shuang-Wen Deng, Jin-Chu Luo, Ming-Xia Li, Li-Ting Zou, Qiu-Gen Zeng, Hong-Feng Chen

**Affiliations:** 1 Key Laboratory of Plant Resources Conservation and Sustainable Utilization, Chinese Academy of Sciences, Guangdong Provincial Key Laboratory of Applied Botany, South China Botanical Garden, Chinese Academy of Sciences, Guangzhou 510650, China South China Botanical Garden, Chinese Academy of Sciences Guangzhou China; 2 University of Chinese Academy of Sciences, Beijing 100049, China University of Chinese Academy of Sciences Beijing China; 3 Zhongkai University of Agriculture and Engineering, Guangzhou 510225, China Zhongkai University of Agriculture and Engineering Guangzhou China; 4 Qingyuan Forestry Bureau, Qingyuan 511515, China Qingyuan Forestry Bureau Qingyuan China

**Keywords:** *Carex* sect. *Siderostictaes.s.*, morphology, new species, taxonomy

## Abstract

*Carexqingyuanensis*, a new species of Cyperaceae from Guangdong Province, China, is described and illustrated. The new species is morphologically similar to *Carexpeliosanthifolia* F. T. Wang & Tang ex P. C. Li, but it can be distinguished by the racemose inflorescence branches appearing single (rarely binate or ternate) (vs. binate or ternate), one (rarely two or three) (vs. 1–3) spiked, male part of linear-cylindrical spikes much longer than the female part (vs. just male part short-cylindrical and slightly longer than female part), style base thickened (vs. not thickened) and perigynium horizontally patent with a short (vs. long and excurved) beak. Phylogenetic analysis, based on the two nuclear DNA regions (ETS 1f and ITS) and three chloroplast DNA regions (*matK*, *ndhF* and *rps16*), suggests that the new species belongs to sect. Siderostictae*s.s.* of subg. Siderosticta and shows a closer phylogenetic relationship to *Carexscaposa* C. B. Clarke.

## ﻿Introduction

*Carex* Linnaeus with ca. 2000 species, is one of the largest angiosperm genera and is distributed almost globally (Dai et al. 2000; Frodin 2004; Global Carex Group 2016). The rapid advancement of sequencing technology has significantly enhanced our comprehension of the relationships within *Carex*. According to the latest phylogenetic study, Villaverde et al. (2020) demonstrated there were six main lineages in *Carex*, identified as the Siderostictae, Schoenoxiphium, Unispicate, Uncinia, Vignea and Core *Carex* clades. Despite advancements, the phylogenetic relationships in *Carex* are not yet sufficient clear for a global reclassification of the genus within a Linnean infrageneric (sectional) framework. Because of this, the Global Carex Group (2021) employed a hybrid approach, utilizing both informally named clades and formally named sections. Their classification system represents the currently understood diversity of *Carex* lineages, encompassing six subgenera, 62 formally designated Linnean sections, and 49 informal groups. In the recently published volume of the *Flora of China* (Dai et al. 2010), a total of 527 species were documented. Since then, nearly 40 new species and two subspecies have been described from China (Jin et al. 2004; Song et al. 2008; Jin 2009; Su 2009a, 2009b; Weng et al. 2009; Jin and Zheng. 2010; Jin et al. 2011, 2012a, 2012b; Zhao et al. 2011; Wang et al. 2012; Yu et al. 2012; Deng 2014; Yang et al. 2014; Jin and Chen 2015; Yang et al. 2015a, 2015b; Yang et al. 2016; Jin 2017; Yang et al. 2017; Lu and Jin 2018; Zhang et al. 2018; Lu et al. 2020; Yang and Liu 2020; Zhang et al. 2021; Li et al. 2022; Lu and Jin 2022; Lu et al. 2022). The new species were mainly discovered in the Provinces of Zhejiang, Guangxi and Anhui.

Many studies provide substantial evidence supporting Carexsubg.Siderosticta as the sister lineage to the remaining taxa within *Carex* (Waterway et al. 2009; Starr et al. 2015; Global Carex Group 2016, 2019; Uzma Jiménez-Mejías et al. 2019; Villaverde et al. 2020). The clade exhibits considerable variability and has a narrow distribution confined primarily to East and Southeast Asia (Global Carex Group 2021).

Carexsect.Siderostictae*s.s.* includes 27 species, in three traditional sections: *Hemiscaposae* (12 species), *Siderostictaes.l.* (13 species) and *Surculosae* (2 species). However, these sections are not supported by the latest phylogenetic hypotheses (Global Carex Group 2021). All species in the traditional sect. Siderostictae*s.l.* and some species in the traditional sect. Hemiscaposae are pink-red at the base of the plant, leaves or bracts (Global Carex Group 2021). Carexsect.Hemiscaposae is unique in *Carex* for its androgynous inflorescence units, leafless pseudolateral culms and pseudopetiolate leaves. The leaves of these species are much wider (up to 12 cm) and show high similarities to *Curculigo* Gaertn (Hypoxidaceae) (Starr et al. 2015; Ford et al. 2017). Moreover, the section is noted especially for its unprecedented variety of inflorescences, ranging from simple to compound (Ford et al. 2017). Eight species have been described within sect. Hemiscaposae, primarily found in broadleaf evergreen forests in southern-central and southeast China (including Taiwan) (Nelmes 1955; Raymond 1959; Yang and Chen 2005; Lunkai et al. 2010). Despite the relatively small number of species, their classification is challenging due to subtle characteristic differences (Ford et al. 2017). Carexsect.Siderostictae*s.s.* is differentiated from the closely related sect. Hypolytroides by the presence of bisexual spikes and leafless fertile culms that seem to emerge laterally from leaf rosettes (Global Carex Group 2021).

During a botanical survey of Bijia Mountain Forest Farm which is located in Qingxin District, Qingyuan City, Guangdong Province, China, covering an area of 2646.11 ha, with an approximate elevation of 1000 m, a new species of Carex in sect. Siderostictae*s.s.* (traditionally placed in sect. Hemiscaposae) (subg. Siderosticta) was discovered in the forest on slopes. A detailed description of the species is provided below.

## ﻿Materials and methods

The material of this new species was collected during the botanical survey conducted at Bijia Mountain Forest Farm. In order to conduct a thorough morphological comparison and phylogenetic analysis, samples of similar species *Carexpeliosanthifolia* and *Carexscaposa* were collected from Shangguchen, Jinxiu County, Guangxi Province, China (110°8′36.69″E, 23°53′42.15″N).

A total of 21 samples representing two sections and three clades were used for molecular phylogenetic analysis, based on the updated infrageneric classification of *Carex* (Global Carex Group 2021) and the sequencing available on GenBank. These sections and clades included Sect. Siderostictae*s.s.* (13 species), Sect. Hypolytroides (two species), Setigera Clade (two species), Decora Clade (two species), Esquiroliana Clade (one species), and *Eriophorumvaginatum* (Table 1). Additionally, this study provided 15 new sequences, which included three species: *C.qingyuanensis*, *C.peliosanthifolia* and *C.scaposa*. The remaining sequences were obtained from the GenBank public database at the National Center for Biotechnology Information (NCBI) (Table 1).

**Table 1.** Genbank numbers for samples used and in combined ITS, ETS 1f, *matK*, *ndhF*, and *rps16* analyses.

Sect.	Species	ITS	ETS 1f	*matK*	*ndhF*	*rps16*
Sect. Siderostictae*s.s.*	*Carexadrienii*	KP273628	KP273594	KP273663	KP273717	KP273771
Sect. Siderostictae*s.s.*	*Carexgeographica*	KX722473	/	KX722479	KX722485	KX722491
Sect. Siderostictae*s.l.*	*Carexglossostigma*	MN762656	/	KP273686	KP273740	/
Sect. Siderostictae*s.l.*	*Carexgrandiligulata*	MW459022	MW458991	MW459089	/	/
Sect. Siderostictae*s.s.*	*Carexkucyniakii*	KP273651	KP273617	KP273695	KP273749	KP273805
Sect. Siderostictae*s.s.*	*Carexpachygyna*	DQ998936	DQ998882	MW459090	/	/
Sect. Siderostictae*s.s.*	*Carexpeliosanthifolia*	OR450685	OR463437	OR464515	OR464518	OR464521
Sect. Siderostictae*s.s.*	*Carexqingyuanensis*	OR450686	OR463436	OR464514	OR464517	OR464520
Sect. Siderostictae*s.s.*	*Carexscaposa*	OR450687	OR463438	OR464516	OR464519	OR464522
Sect. Siderostictae*s.l.*	*Carexsiderosticta*	KP273658	KP273624	KJ513592	KJ513499	KP273817
Sect. Siderostictae*s.s.*	*Carexthinii*	KX722474	KX722468	KX722480	KX722486	KX722492
Sect. Siderostictae*s.s.*	*Carextsiangii*	KU496610	KU377556	KU496590	/	/
Sect. Siderostictae*s.l.*	*Carexwuyishanensis*	MW459024	MW458993	MW459091	/	/
sect. Hypolytroides	*Carexhypolytroides*	KP273647	KP273612	KP273690	KP273744	KP273800
sect. Hypolytroides	*Carexmoupinensis*	KP273653	KP273619	KP273699	KP273753	KP273809
Setigera Clade	*Carexbaccans*	KP273632	KP273598	KP273669	KP273723	KP273778
Setigera Clade	*Carexmyosurus*	KP273654	KP273620	KP273700	KP273754	KP273810
Decora Clade	*Carexcruciata*	KP273637	KP273603	KP273676	KP273730	KP273787
Decora Clade	*Carexfilicina*	KP273642	KP273608	KP273682	KP273736	KP273793
Esquiroliana Clade	*Carexesquiroliana*	MN762053	MN761064	MN763585	/	/
Outgroup	*Eriophorumvaginatum*	AH012952.2	AH012952.2	KJ513615	KJ513522	KP273830

Two nuclear DNA regions (ETS 1f and ITS) and three chloroplast DNA regions (*matK*, *ndhF* and *rps16*) (at least three genes) were used for the phylogenetic analysis. The amplified primers followed Starr et al. (2003) for ETS 1f, White et al. (1990) and Blattner (1999) for ITS, Gilmour et al. (2013) for *matK* and *ndhF* and Starr et al. (2015) for *rps16*.

The sequences were aligned using the online version of MAFFT (Katoh et al. 2019). The ETS 1f, ITS, *matK*, *ndhF* and *rps16* were combined in eight Sequence Matrix (combined nDNA-cpDNA; combined nDNA; combined cpDNA; ETS 1f; ITS; *matK*; *ndhF*; and *rps16*). PhyloSuite (Zhang et al. 2020) software was used to generate the Maximum Likelihood (ML) tree and Bayesian (BI) trees, with *Eriophorumvaginatum* set as the outgroup (Starr et al. 2015). ML analysis was conducted using IQ-TREE (Nguyen et al. 2015) with 5000 standard non-parametric bootstrap replicates. BI analysis was performed using MrBayes 3.2.6 (Ronquist et al. 2012), two independent parallel chains and 5,000,000 generations with sampling once every 100 generations. The first 25% of trees from all runs were discarded as burn-in. Finally, ITOL (Ivica and Peer 2019) software was used for tree visualisation and refinement.

## ﻿Taxonomic treatment

### ﻿ Carex qingyuanensis

Taxon classificationPlantae

Y.L. Li & H.F. Chen sp. nov.

B20A9870-240D-50CC-B3F4-2FF3385CFFB6

urn:lsid:ipni.org:names:77340693-1

#### Type.

**Figure 1.**
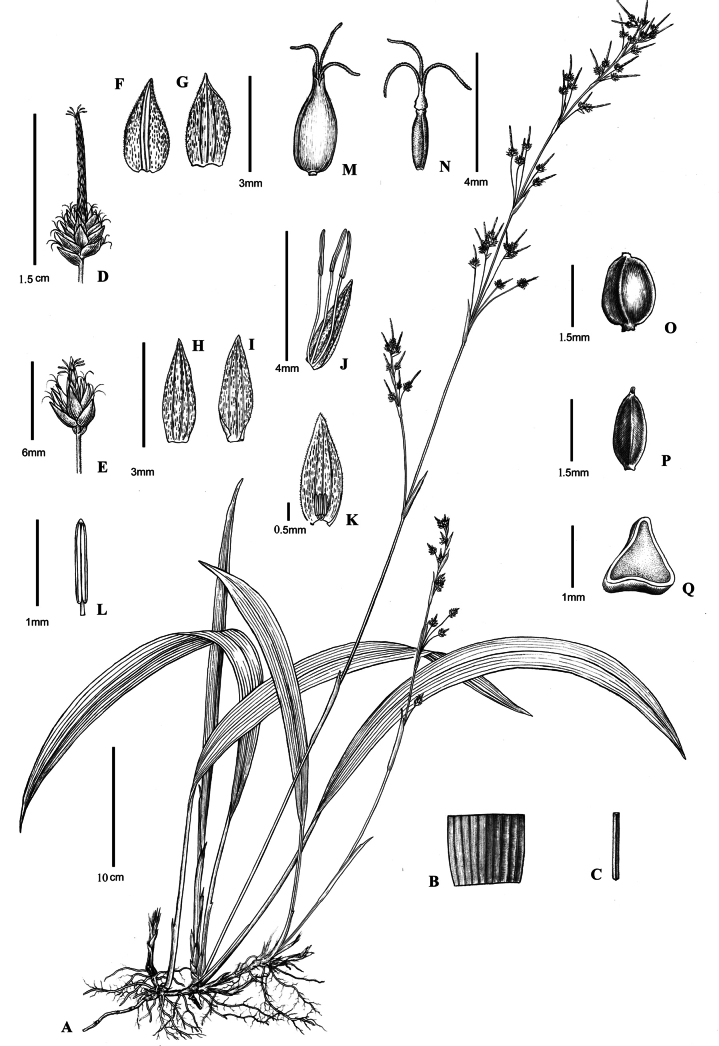
**A***Carexqingyuanensis***B** abaxial and adaxial surface of leaf blade **C** culm **D, E** spikes **F** adaxial surface of pistillate glume **G** abaxial surface of pistillate glume **H** adaxial surface of staminate glume **I** abaxial surface of staminate glume **J, K** staminate flower **L** anther **M** perigynium **N** stigmas **O, P** nutlets **Q** the transection of nutlet. Drawn by Mrs. Liu Yunxiao based on *Li Yali & Chen Hongfeng LYL0012*.

**Figure 2.**
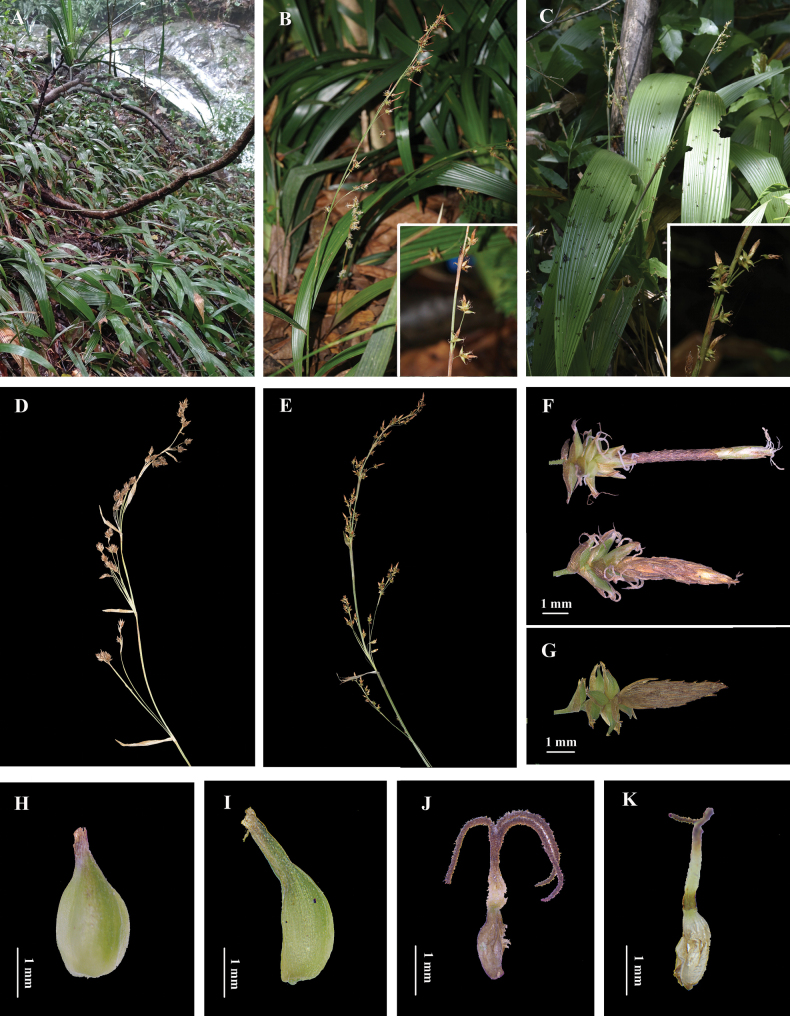
**A** habitat of *Carexqingyuanensis***B** infructescence of *C.qingyuanensis***C** infructescence of *C.peliosanthifolia***D** inflorescence of *C.qingyuanensis***E** inflorescence of *C.peliosanthifolia***F** spikes of *C.qingyuanensis***G** spike of *C.peliosanthifolia***H** short beak of *C.qingyuanensis***I** long beak of *C.peliosanthifolia***J** immature nutlets + base thickened style of *C.qingyuanensis***K** immature nutlets + unthickened style of *C.peliosanthifolia*.

China. Guangdong Province, Qingyuan City, Qingxin District, Bijia Mountain Forest Farm, 23°49′45″N, 113°03′07″E, 600 m elev., in the forest, on the rocks, 18 November 2022, *Li Yali* & *Chen Hongfeng LYL0012* (holotype: IBSC; isotype: IBSC) (Figs 1, 2).

#### Diagnosis.

The new species is similar to *Carexpeliosanthifolia* F. T. Wang & Tang ex P. C. Li, but differs by having inflorescence branches racemose, single (rarely binate or ternate), one (rarely two or three) spiked, (vs. binate or ternate, one-three spiked), male part of spikes short-cylindrical or linear-cylindrical and slightly or much longer than female part (vs. just male part short-cylindrical and slightly longer than female part); style base thickened (vs. not thickened); beak short and slightly curved (vs. long and excurved).

#### Description.

Perennial herbs. Rhizome stoloniferous, woody. Culms lateral, trigonous, loosely pubescent, base with brown sheaths, 15–60 cm tall. Leaves basal and cauline; basal leaves 1(2–3) tufted on rhizome node; petiole 15–25 cm long, folded, glabrous; blades narrowly elliptical, 18–30 × 2.5–5 cm, glabrous or scabrid on abaxial veins, replicate, base attenuate, apex acuminate; cauline leaves pale greenish-white with dense brown spots and short lines, spathe-like, pubescent. Involucral bracts spathe-like. Panicle compound; inflorescence branches racemose, 5–8 branched, single (rarely binate or ternate), 1 (rarely 2 or 3) spiked; peduncles of inflorescence branches tenuous, 0.5–8 cm long, densely pubescent; bractlets glume-like, ovate-oblong, ca. 3.5 mm long. Spikes bisexual, densely flowered, androgynous; spikes 6–15 mm long, male part short-cylindrical to linear-cylindrical, slightly or much longer than female part, with ca. 15–40 staminate flowers; female part with 6–25 pistillate flowers. Staminate glumes pale yellow laterally with dense spots and short lines, pale green at middle, ovate–lanceolate, ca. 3 mm long, membranous, 3–veined, apex acuminate; pistillate glumes similar to staminate glumes. stamens 3, filaments basally connate, 0.5–3 mm long, longer or remarkably shorter than staminate glumes; anther yellow, 1 × 0.2 mm, pollen 0.2 mm wide; perigynium pale yellowish-white with brown spots and short lines, horizontally patent, elliptical, trigonous, 2.6–3.3 mm long, membranous, glabrous, with many raised veins, base subrounded, apex attenuate into a slightly curved beak, ca. 0.5 mm long. Nutlets brown at maturity, tightly enveloped, elliptical, trigonous or base obliquely truncate, 1–2 mm long; style suberect, base thickened; stigmas 3.

#### Phenology.

Flowering from August to November. Fruiting from December to February.

#### Etymology.

The term “qingyuanensis” originates from the location where the type specimen was collected.

#### Distribution and habitat.

*Carexqingyuanensis* is known only from Bijia Mountain Forest Farm, Qingxin District, Qingyuan City, Guangdong Province, China. It grows on rocky terrain within the forest at an elevation of 600 m (Fig. 2A).

#### Conservation status.

Currently, *Carexqingyuanensis* is only known from its type locality, Bijia Mountain Forest Farm, Qingxin District, Qingyaun City, Guangdong Province, China, which covers an area of 2646.11 ha. Based on the IUCN Red List Criteria (IUCN 2022), the species could be assessed as Endangered (EN) or Vulnerable (VU). However, at present, it is more appropriate to classify it as Data Deficient (DD) due to the absence of field surveys conducted on the populations of this species.

#### Additional specimens examined.

China. Guangdong: Qingyuan City, Qingxin District, Bijia Mountain Forest Farm, 18 November 2022, *Li Yali & Chen Hongfeng LYL0013* (IBSC); Qingyuan City, Qingxin District, Bijia Mountain Forest Farm, 1 December 2022, *Li Yali & Chen Hongfeng LYL0014* (IBSC).

## ﻿Results

**Figure 3.**
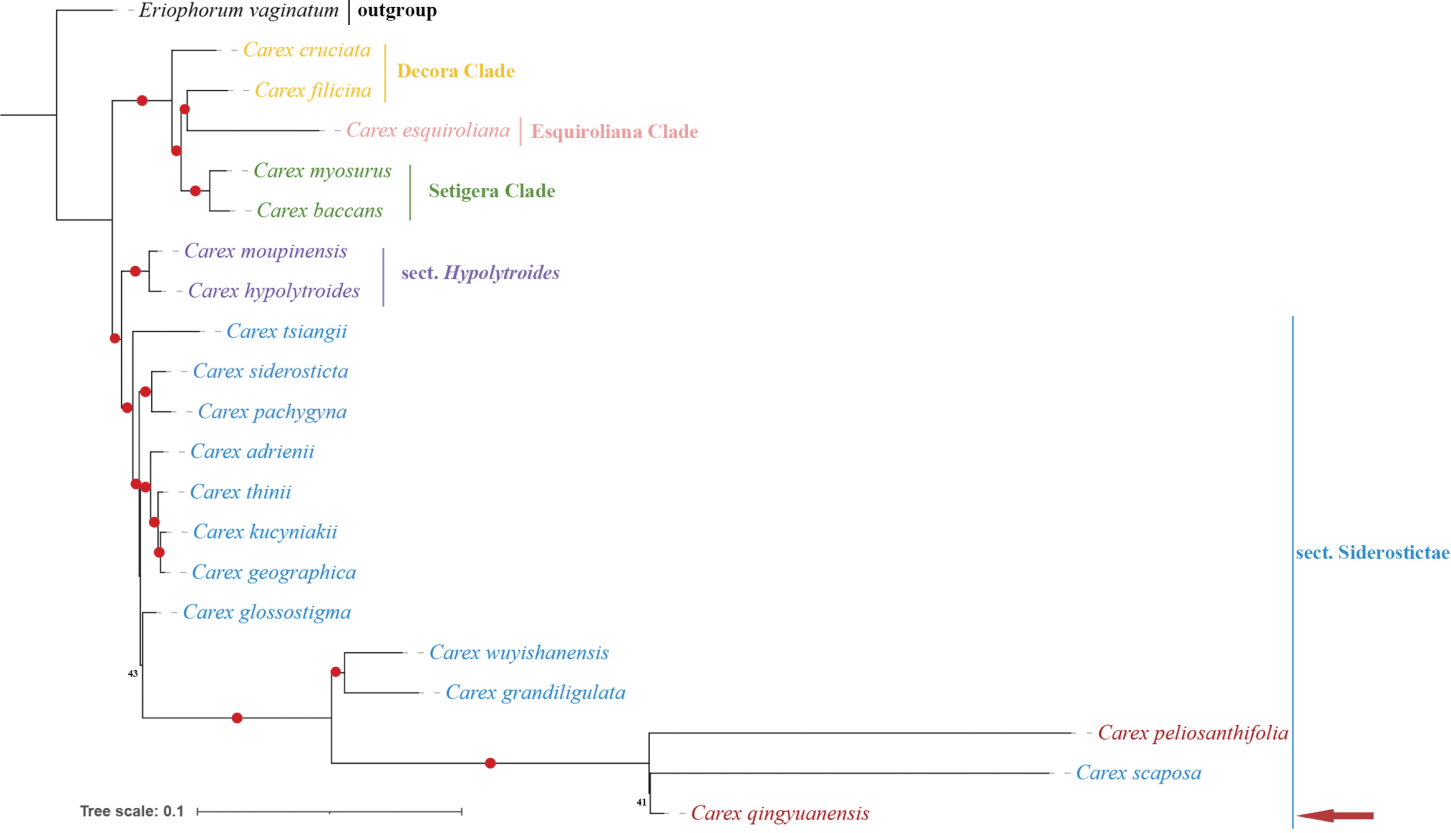
Phylogenetic relationships of 21 species by combined nDNA-cpDNA matrices. *Eriophorumvaginatum* was set as outgroup. The phylogenetic tree was constructed by MrBayes (BI). The bootstrap values are represented at nodes, red circles indicate bootstrap values of 90–100%, while the rest are marked with numbers.

The phylogenetic trees inferred from ML and BI shared an identical topology, while BI showed higher support values. Matrices of combined nDNA-cpDNA (Fig. 3) and combined nDNA (Suppl. material 1: fig. S1) yielded similar topologies, but slight differences in phylogenetic relationships within the sect. Siderostictae*s.s.* and the relationship between sect. Hypolytroides and sect. Siderostictae*s.s.*. Except for combined cpDNA (Suppl. material 1: fig. S4), *matK* (Suppl. material 1: fig. S5), and *ndhF* (Suppl. material 1: fig. S6) matrices, species within sect. Siderostictae*s.s.* consistently formed a single clade (Fig. 1, Suppl. material 1: figs S1–S3, S7). As indicated by the study conducted by Villaverde et al. (2020) and Global Carex Group (2021), analyses performed on the combined nDNA-cpDNA matrices effectively illustrate the phylogenetic relationship among 21 species (Fig. 3). *Carexbaccans*, *C.myosurus*, *C.cruciata*, *C.filicina*, and *C.esquiroliana* are part of the core Carex Clade, while species in sect. Hypolytroides and sect. Siderostictae*s.s.* (Global Carex Group 2021) are categorized under the *Siderosticta* clade according to Villaverde et al. (2020). The phylogenetic trees (Fig. 3) strongly reinforce the intrageneric relationships within *Siderosticta* clade and core Carex Clade. Furthermore, sect. Hypolytroides is identified as sister group to sect. Siderostictae*s.s.* (Fig. 3) remains consistent with the findings of both Villaverde et al. (2020) and Global Carex Group (2021) studies. Based on the topology, among the 13 species within sect. Siderostictae*s.s.*, *C.qingyuanensis* shows closer phylogenetic relationship to *C.scaposa* rather than to *C.peliosanthifolia*.

## ﻿Discussion

According to the classification by Dai et al. (2000) and Global Carex Group (2021), *Carexqingyuanensis* belongs to sect. Siderostictae*s.s.* (traditionally placed in sect. Hemiscaposae) (subg. Siderosticta). Morphologically, it shares similarities with *C.peliosanthifolia* in having basal leaves tufted, fan-shaped; panicle compound, branches not only single and perigynium with many raised veins, but differs by having inflorescence branches racemose, single (rarely binate or ternate), one (rarely two or three) spiked, (vs. binate or ternate, 1–3-spiked), male part of spikes short-cylindrical or linear-cylindrical and slightly or much longer than the female part (vs. just male part short-cylindrical and slightly longer than female part); style base thickened (vs. not thickened) and beak short and slightly curved (vs. long and excurved) (Dai et al. 2000, 2010). Morphological comparisons of *C.qingyuanensis* and *C.peliosanthifolia* are summarised in Table 2. Furthermore, an updated identification key to Chinese species in the traditionally sect. Hemiscaposae is provided below.

**Table 2.** Morphological comparison among *Carexqingyuanensis*, *Carexpeliosanthifolia*, and *Carexscaposa*.

Character	*C.qingyuanensis*	*C.peliosanthifolia*	*C.scaposa*
Inflorescence branches	Single (rarely binate or ternate), one (rarely two or three) spiked	Binate or ternate, one-three spiked;	Panicle compound
Spikes	Long, male part short-cylindrical or linear-cylindrical and slightly or much longer than female part	Short, male part short-cylindrical and slightly longer than female part	Male part of spike linear-lanceolate, usually shorter than female part
Pistil	Style base thickened	Style base not thickened	Style base not or slightly thickened
Perigynium	Apex attenuate into a slightly curved short beak	Apex attenuate into an excurved long beak	Apex contracted gradually into beak of medium length

### ﻿Key to the species of Carexsect.Siderostictae*s.s.* (traditional in sect. Hemiscaposae) in China

**Table:** 

1	Ten or more spikes on each branch; leaves flat; perigynium with 2 lateral veins on adaxial surface	**2**
–	Fewer than 10 spikes on each branch; leaves fan-shaped; perigynium with many raised veins	**7**
2	Panicle compound, several branched; culms rigid	**3**
–	Panicle simple, only one terminal branched or 1–2 lateral branched; culms flaccid	**6**
3	Inflorescence branches paniculate, 10–20 spikes; male part of spikes oblong-cylindrical	***C.scaposa***
–	Inflorescence branches subcorymbose, a few spikes; male part of spike circular or oblong	**4**
4	Leaves linear or linear-oblanceolate; perigynium ovate, beak ca. 1/4 length of perigynium	***C.liouana***
–	Leaves narrowly elliptical to elliptical-linear; perigynium elliptical, beak slightly shorter than 1/2 length of perigynium	**5**
5	Leaves margin glabrous, scabrid abaxially; petiole glabrous; nutlets ovate	***C.adrienii***
–	Leaves margin densely ciliate, glabrous on both surfaces or scabrid or densely hairy abaxially; petiole hairy; nutlets elliptical	***C.densifimbriata***
6	Leaves narrowly elliptical to linear-elliptical, margins densely replicate; male part of spike circular to oblong, 2.5–4 mm long, ca. 3 mm wide; culms loosely hairy	***C.lingii***
–	Leaves band shape, margins flat; male part of spikes circular-cylindrical, 3–5 mm long, ca. 1 mm wide; culms loosely hairy and then glabrescent	***C.ypsilandrifolia***
7	Branches single, rarely binate; basal leaf single	***C.kucyniakii***
–	Branches not only single; basal leaves 1–3 tufted	**8**
8	Branches binate or ternate. male part of spikes short-cylindrical	***C.peliosanthifolia***
–	Branches single (binate or ternate). Male part of spikes short-cylindrical or linear-cylindrical	***C.qingyuanensis***

## Supplementary Material

XML Treatment for Carex qingyuanensis

## ﻿Additional information

### Author ORCIDs

Ya-Li Li https://orcid.org/0000-0003-4667-5241

Shuang-Wen Deng https://orcid.org/0000-0002-1595-2974

Hong-Feng Chen https://orcid.org/0000-0002-8415-3260

### Data availability

All of the data that support the findings of this study are available in the main text or Supplementary Information.

## Supplementary materials

Supplementary material 1 Phylogenetic relationships Data type pdf Explanation note **fig. S1.** Phylogenetic relationships of 21 species by combined nDNA matrices. *Eriophorumvaginatum* was set as the outgroup. The phylogenetic tree was constructed by MrBayes (BI). The bootstrap values are represented at nodes, red circles indicate bootstrap values of 90–100, while the rest are marked with numbers. The species marked in yellow belong to the Decora Clade, the ones in pink belong to the Esquiroliana Clade, the ones in green belong to the Setigera Clade, the ones in purple belong to sect. Hypolytroides, and those in blue belong to sect. Siderostictae*s.s.*. The ones highlighted in red belong to sect. Siderostictae*s.s.* and share similar morphological characteristics. **fig. S2.** Phylogenetic relationships of 19 species by ETS matrices. The notes are same as fig. S1. **fig. S3.** Phylogenetic relationships of 21 species by ITS matrices. The notes are same as fig. S1. **fig. S4.** Phylogenetic relationships of 21 species by combined cpDNA matrices. The notes are same as fig. S1. **fig. S5.** Phylogenetic relationships of 21 species by *matK* matrices. The notes are the same as fig. S1. **fig. S6.** Phylogenetic relationships of 16 species by *ndhF* matrices. The notes are the same as fig. S1. **fig. S7.** Phylogenetic relationships of 15 species by *rps16* matrices. The notes are the same as fig. S1. https://binary.pensoft.net/file/1032531This dataset is made available under the Open Database License (http://opendatacommons.org/licenses/odbl/1.0/). The Open Database License (ODbL) is a license agreement intended to allow users to freely share, modify, and use this Dataset while maintaining this same freedom for others, provided that the original source and author(s) are credited.Ya-Li Li, Shuang-Wen Deng, Jin-Chu Luo, Ming-Xia Li, Li-Ting Zou, Qiu-Gen Zeng, Hong-Feng Chen
