# Supplementary material for: ﻿Carexqingyuanensis (Cyperaceae), a new species from Guangdong, China
Source: PhytoKeys. 2024 Apr 25;241:155–68. doi: 10.3897/phytokeys.241.117734 (PMC11066503; doi:10.3897/phytokeys.241.117734)
Supplement: Supplementary material 1 — Phylogenetic relationships [file phytokeys-241-155_article-117734__-s001.pdf]

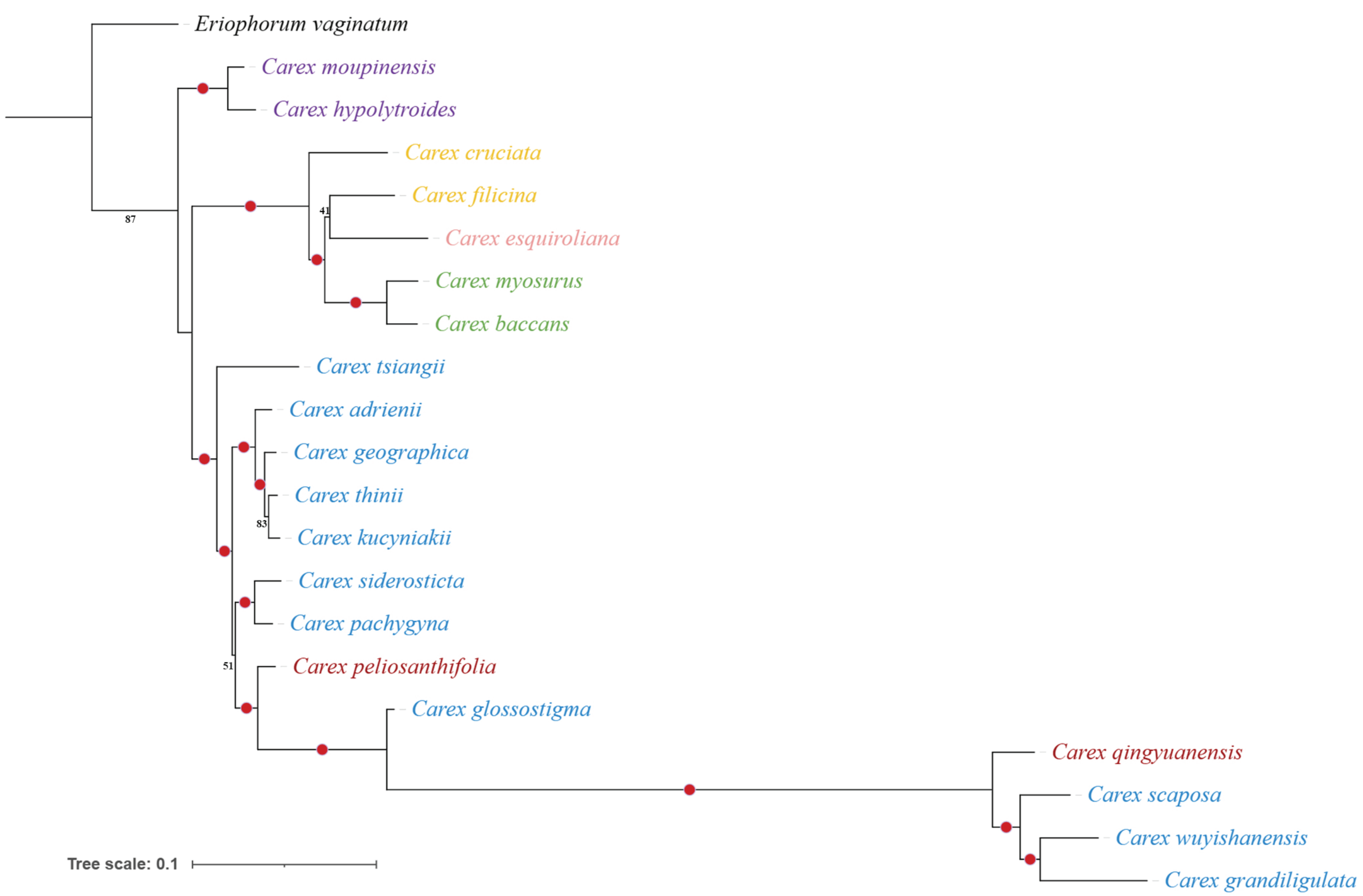

Figure S1. Phylogenetic relationships of 21 species by combined nDNA matrices. *Eriphorum vaginatum* was set as the out-group. The phylogenetic tree was constructed by MrBayes (BI). The bootstrap values are represented at nodes, red circles indicate bootstrap values of 90-100, while the rest are marked with numbers. The species marked in yellow belong to the Decora Clade, the ones in pink belong to the Esquiroliana Clade, the ones in green belong to the Setigera Clade, the ones in purple belong to sect. *Hypolytroides*, and those in blue belong to sect. *Siderostictae s.s.*. The ones highlighted in red belong to sect. *Siderostictae s.s.* and share similar morphological characteristics

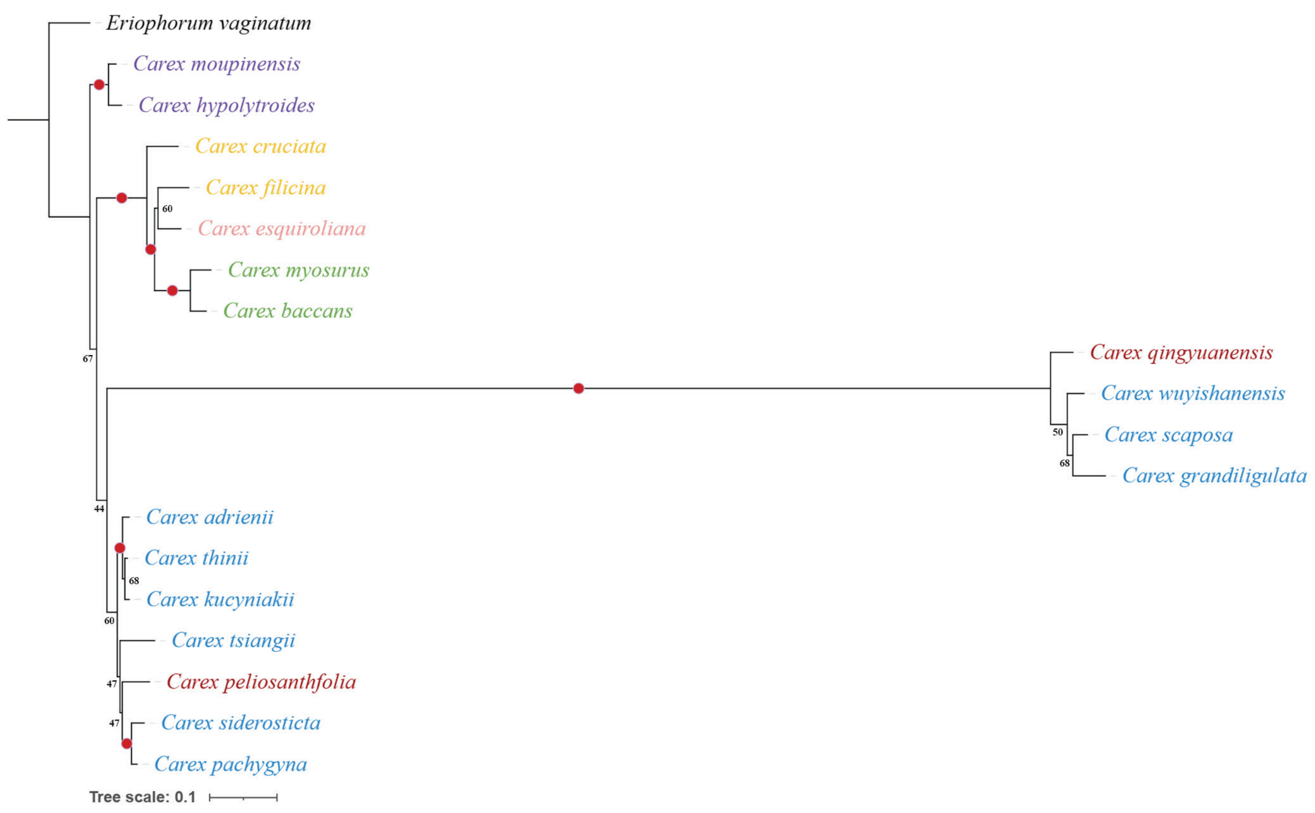

Figure S2. Phylogenetic relationships of 19 species by ETS matrices. The notes are same as Fig. S1.

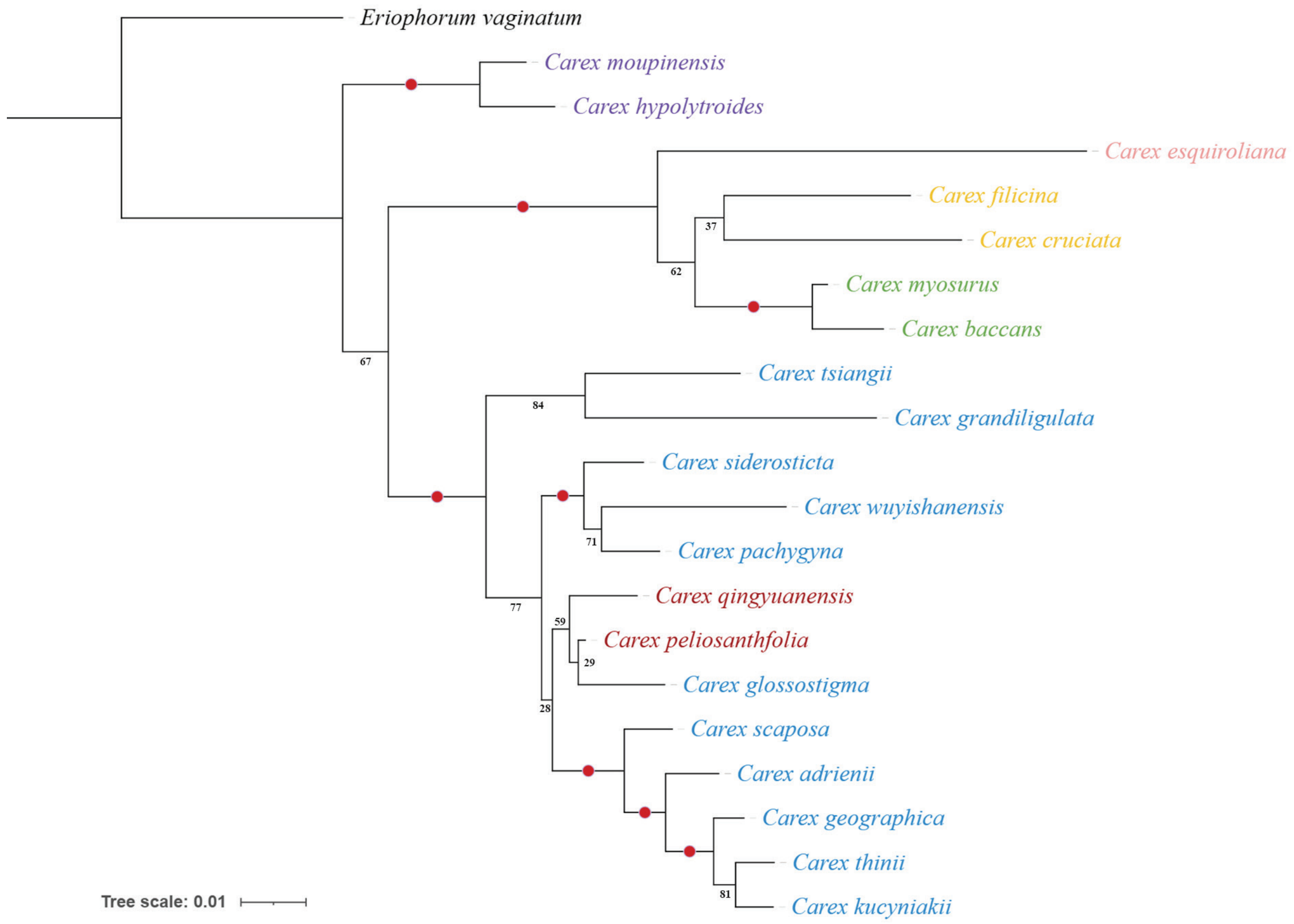

Figure S3. Phylogenetic relationships of 21 species by ITS matrices. The notes are same as Fig. S1.

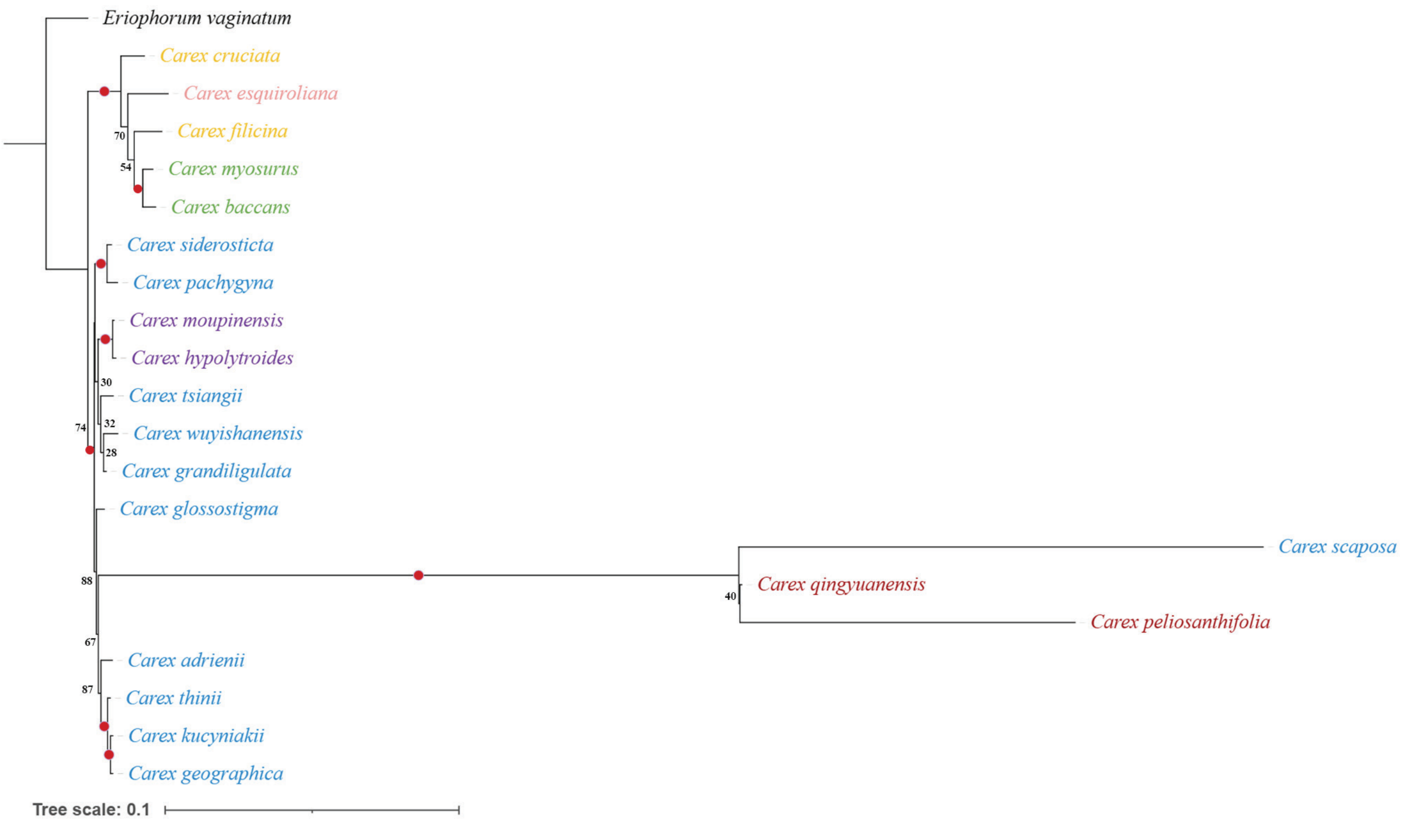

Figure S4. Phylogenetic relationships of 21 species by combined cpDNA matrices. The notes are same as Fig. S1.

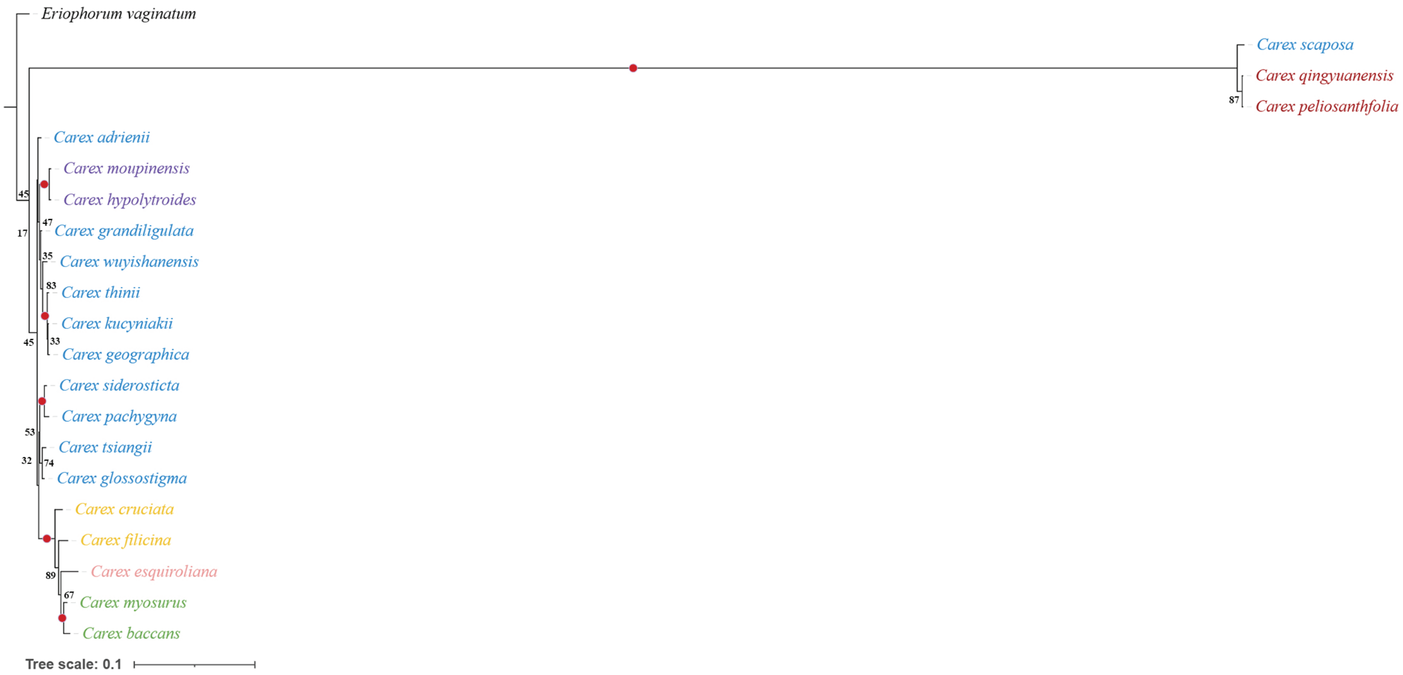

Figure S5. Phylogenetic relationships of 21 species by *matK* matrices. The notes are the same as Fig. S1.

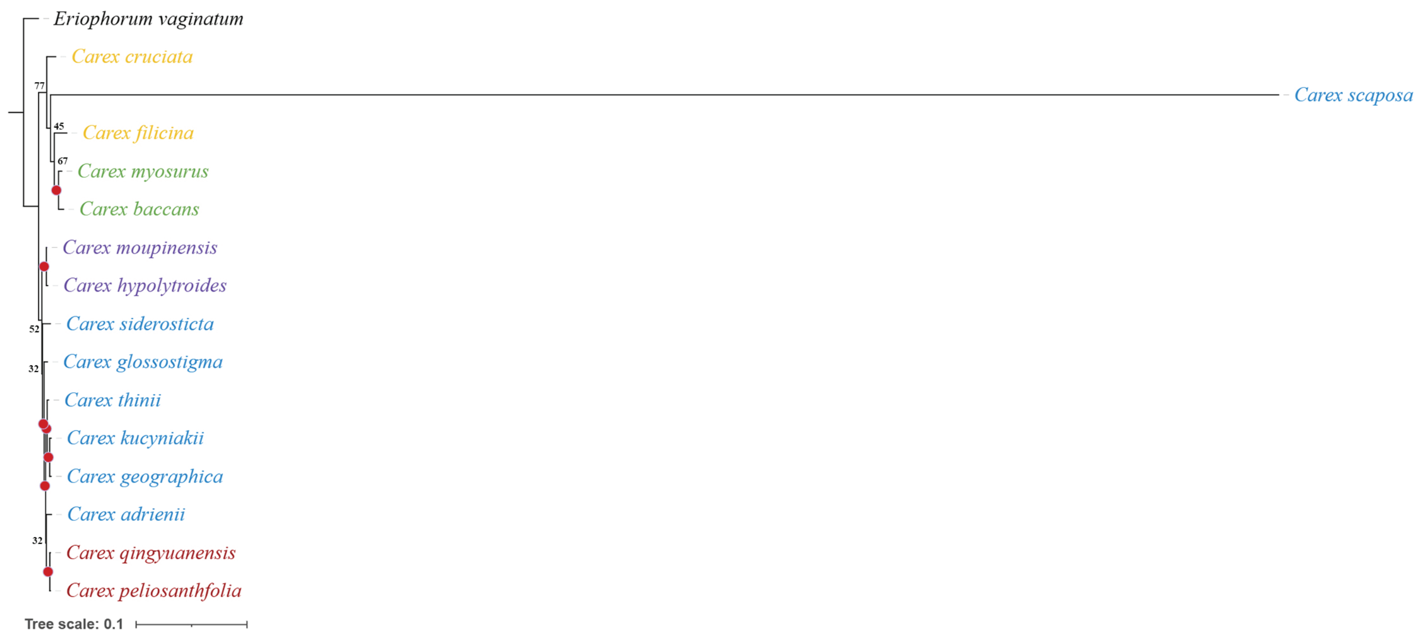

Figure S6. Phylogenetic relationships of 16 species by *ndhF* matrices. The notes are the same as Fig. S1.

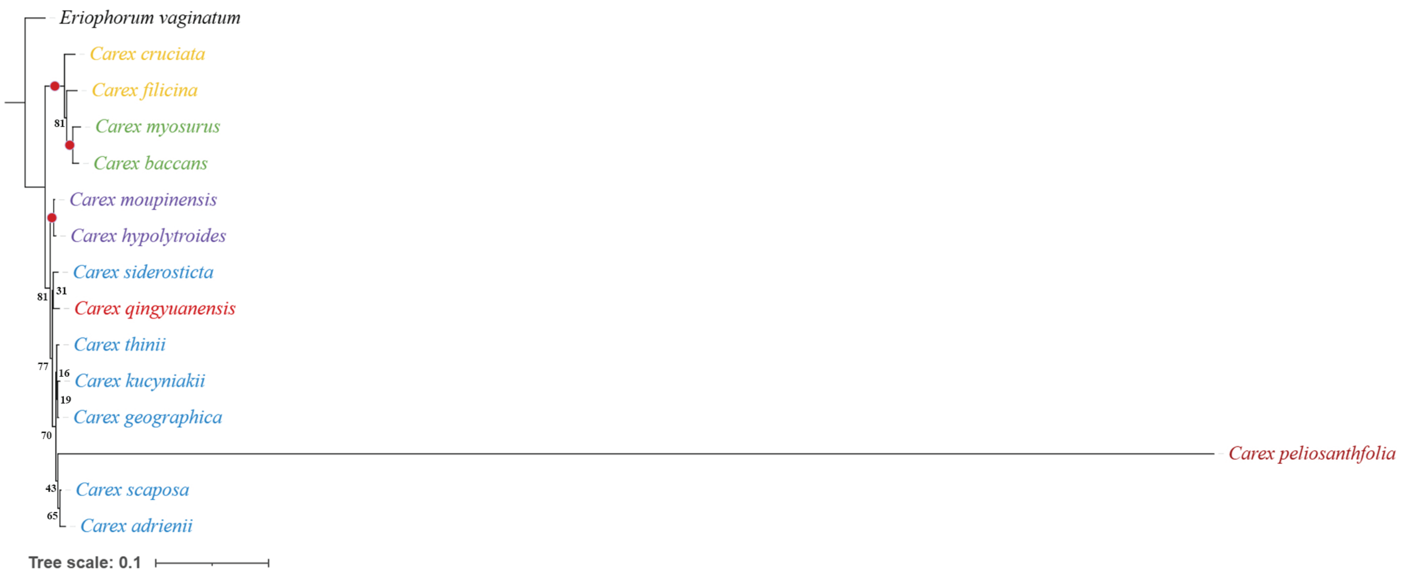

Figure S7. Phylogenetic relationships of 15 species by *rps16* matrices. The notes are the same as Fig. S1.
